# The DMC-Behavior Platform: An Open-Source Framework for Auditory-Guided Perceptual Decision-Making in Head-Fixed Mice

**DOI:** 10.1523/ENEURO.0457-24.2025

**Published:** 2025-04-08

**Authors:** Felix Jung, Xiao Cao, Loran Heymans, Marie Carlén

**Affiliations:** Department of Neuroscience, Karolinska Institutet, Stockholm 171 77, Sweden

**Keywords:** auditory, head-fixed behaviors, perceptual decision-making, Raspberry Pi, standardization

## Abstract

Perceptual decision-making describes the process of selecting an appropriate action based on the sensory information present in the immediate environment and is hence an omnipresent factor in the life of animals. In preclinical research, a widespread approach to study the neuronal correlates of perceptual decision-making is to record (and manipulate) neuronal activity in head-fixed mice performing behavioral tasks. In contrast to the technologies used to record/manipulate neuronal activity, standardization of the behavioral training of mice is generally neglected, a circumstance that is particularly true for behavioral tasks involving auditory stimuli. Here, we present the DMC-Behavior Platform, an open-source, cost-efficient framework for training head-fixed mice in perceptual decision-making tasks involving auditory stimuli. Combining the DMC-Behavior Platform with strategies to record and manipulate neuronal activity offers many opportunities to test hypotheses on the neuronal underpinnings of cognitive processes. We demonstrate the utility of the platform by training mice on auditory variants of three behavioral tasks commonly used to study perceptual decision-making: a detection, a Go/NoGo, and a two-alternative forced choice task. The presented work establishes the seamless integration and synchronization of external devices to record neuronal activity, task-related, and nontask-related behavioral variables. Our platform provides a valuable tool for more standardized and reproducible investigations into the neuronal circuits underlying auditory-guided decision-making.

## Graphical abstract

**Figure:**
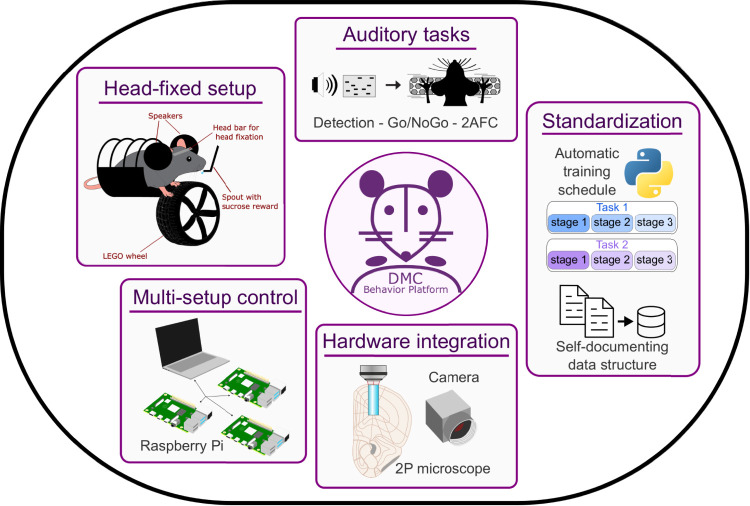
Visual Abstract

## Significance Statement

Technologies to record large populations of neurons embedded in brain-wide neuronal circuits are increasingly commonplace in the neuroscientific community, often with a focus on head-fixed mice performing behavioral tasks. Most laboratories rely on in-house development of customized solutions to implement head-fixed behaviors which limits standardization and replicability of results. Addressing this bottleneck, we present the DMC-Behavior Platform, a low-cost and open-source framework for standardized training of head-fixed mice in behavioral tasks using auditory stimuli. We demonstrate the platform's effortless integration of external devices to record neuronal activity highlighting the potential of its broad utilization.

## Introduction

Perceptual decision-making is an omnipresent factor in the life of animals and a critical determinant for survival. Understanding how these cognitive operations are implemented in neuronal circuits is a central goal of current systems neuroscience research (Carandini and Churchland, 2013). In the last two decades, significant advancements in genetic engineering and in neuroscience technology have placed mice at the forefront of the fields’ research endeavors (Carandini and Churchland, 2013; Luo et al., 2018; Le Merre et al., 2021). An increasing number of transgenic Cre-mouse lines allows the utilization of a similarly ever-increasing number of tools to manipulate (e.g., chemo-/optogenetically; Deisseroth, 2015; Kang et al., 2023) or record neuronal activity (e.g., optically or electrophysiologically; Urai et al., 2022) from defined circuit components. Although ground-breaking developments in using large-scale recording devices in freely moving mice have been made recently (Ghosh et al., 2011; Steinmetz et al., 2021; Zong et al., 2022), most current large-scale neuronal recordings are still performed in head-fixed mice. Alongside the technological developments has grown the need for cost-efficient, open-source platforms to establish standardized, reproducible, and complex behavioral tasks in (head-fixed) mice (Burgess et al., 2017; White et al., 2019; The International Brain Laboratory et al., 2021; Gordon-Fennell et al., 2023).

In line with this demand, we present a cost-efficient (∼1,300 EUR), open-source framework (the DMC-Behavior Platform) optimized to studying decision-making processes using auditory stimuli (i.e., auditory decision-making) in head-fixed mice. Behavior is guided by liquid rewards, and the animals report their decisions by turning a steering wheel positioned under their forepaws (adapted from the International Brain Laboratory (IBL) pipeline; The International Brain Laboratory et al., 2021; Movie 1; Extended Data Video 1). We demonstrate the utilization of the DMC-Behavior Platform by applying auditory variants of three commonly used decision-making paradigms (Carandini and Churchland, 2013): a detection task, a Go/NoGo task, and a two-alternative forced choice (2AFC) task. Detailed protocols are included for user-friendly assembly and installation of the platform for running and/or customization of the presented behavioral tasks (https://github.com/hejDMC/dmc-behavior). Implementation of new tasks for studying decision-making using auditory stimuli is also feasible. Further, the DMC-Behavior Platform includes automatic tracking of training progression and a system for organized behavioral data storage to standardize training and data acquisition. We exemplify the platform's seamless integration with external hardware devices recording behavioral variables and neuronal activity from task performing mice. For this, the platform generates signals to control an external video camera recording motoric activity and detects synchronization signals from a 2-photon calcium imaging microscope.

**Movie 1:** [View online] Head-fixed task behaving animal with DLC tracking of tongue and forepaws.

## Materials and Methods

### DMC-Behavior Platform

**Figure 1.**
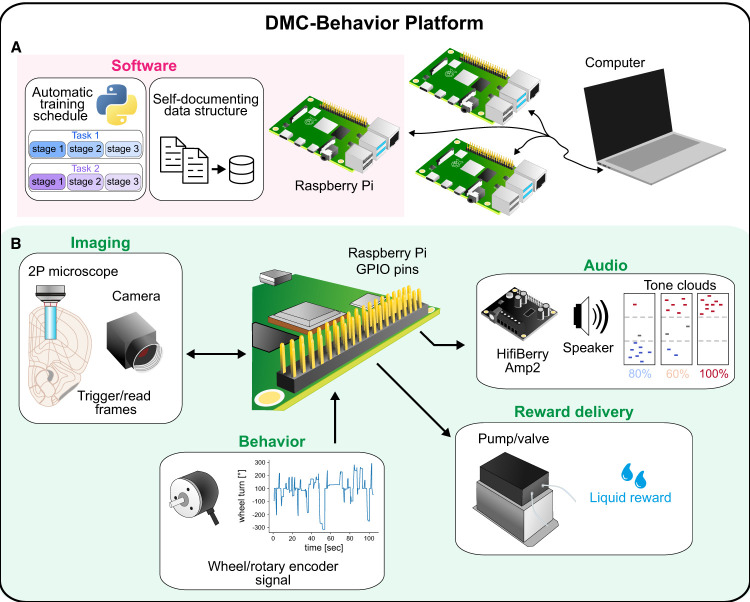
Components of the DMC-Behavior Platform. ***A***, The DMC-Behavior Platform is organized around Python implementations run on a single-board computer (Raspberry Pi). The DMC-Behavior Platform allows programming of a variety of auditory behavioral tasks with automated, staged training procedures, and storage of detailed session data without user interference. The Raspberry Pi is remotely accessed and controlled from a computer via the SSH protocol, with simultaneous control of multiple Raspberry Pi's being feasible. ***B***, The Raspberry Pi's General-Purpose Input/Output (GPIO) pins interface with different hardware components. Auditory signals (tones/noises/tone clouds, etc.) are *de novo* generated for each trial using Python (44.1–192 kHz sampling rate). The auditory signals are reconstructed and amplified using a HifiBerry Amp2 board mounted on top of the Raspberry Pi and played by speakers. Rotary encoder signals (encoding the position of the steering wheel) are used for computing the animal's wheel movement and stored for offline analysis. Reward delivery systems (pumps/valves) are controlled by TTL output signals sent via the Raspberry Pi's GPIO pins. The DMC-Behavior Platform can record signals from, or generate signals controlling, external hardware devices recording motoric expressions or neuronal activity (e.g., frame pulses from imaging devices). In the same vein light sources for optogenetic manipulations can be incorporated, and imaging of neuronal activity be exchanged for electrophysiological recordings (data not shown).

The DMC-Behavior Platform integrates soft- and hardware components for running auditory behavioral tasks for head-fixed mice (Fig. 1). A complete list of hardware parts, a detailed protocol for assembling the setup and installing the software, all code, and background information on code and data organization are provided online. The code/software described in the paper is freely available online at https://github.com/hejDMC/dmc-behavior. The code is available as Extended Data 1.

10.1523/ENEURO.0457-24.2025.d1 Extended data 1 DMC-Behavior repository. Download Extended data 1, ZIP file.

In brief, a single-board computer (Raspberry Pi 4B, Raspberry Pi Foundation) is used for each behavioral setup to run the software implementations (Python). Each Raspberry Pi is remotely accessed and controlled from a central computer using the Secure Shell (SSH) protocol, allowing for parallel control of multiple behavioral setups (Fig. 1*A*). All hardware components are connected to the Raspberry Pi's general-purpose input/output (GPIO) pins allowing reading information from and/or controlling the respective hardware (Fig. 1*B*).

As in the IBL task (The International Brain Laboratory et al., 2021), the animals indicate their choices by turning a steering wheel (LEGO) positioned under their forepaws. The steering wheel's position is detected using a high-resolution rotary encoder (05.2400.1122.1024, Kübler) and processed online. The DMC-Behavior Platform utilizes auditory stimuli presented via two speakers (K 28 WP 8 Ohm, Visaton) positioned symmetrically on either side of the animal's head. Auditory stimuli (e.g., tones/noises/tone clouds) are reconstructed and amplified using an HifiBerry Amp2 digital-to-analog converter (DAC; HiFiBerry). Liquid rewards are delivered by a pump (Campden Instruments) controlled by standard TTL pulses. Additional GPIO channels of the board can be used to receive or send signals to synchronize and control external hardware components such as cameras, lasers, or electrophysiological/imaging-recording systems.

The software components are adapted from principles used in the Autopilot framework (Saunders and Wehr, 2019) and enable the user to program a variety of auditory tasks with ranging levels of difficulty. The software entails automated tracking of training stage progression and data storage, minimizing user interference and workload.

### Animals

All procedures and experiments on animals were performed according to the guidelines of the Stockholm Municipal Committee for animal experiments and the Karolinska Institutet in Sweden (approval number 7362-2019). A complete list of animals is presented in Table 1. Animals were housed in groups with up to four animals per cage, in a temperature- (23°C) and humidity-controlled (55%) environment in standard cages on a 12 h light/dark cycle. Food and water were available *ad libitum*, except during water restriction periods.

**Table 1.** List of animals included in the manuscript

Animal ID	Task	Sex	Genotype	Age at surgery	Age at beginning of training	Data shown
DP-374	Detection	Female	C57BL/6J	8 w	9 w	Fig. 2*E*,*G*
DP-375	Detection	Female	C57BL/6J	8 w	9 w	Fig. 2*E*,*G*
DP-376	Detection	Female	C57BL/6J	8 w	9 w	Fig. 2*B–G*
DP-364	Go/NoGo	Female	C57BL/6J	13 w	14 w	Fig. 3*B–G*,*I*
DP-365	Go/NoGo	Female	C57BL/6J	13 w	14 w	Fig. 3*I*
DP-366	Go/NoGo	Female	C57BL/6J	13 w	14 w	Fig. 3*I*
DP-371	Go/NoGo	Male	C57BL/6J	13 w	14 w	Fig. 3*H*,*I*
DP-377	2AFC	Male	C57BL/6J	8 w	12 w	Fig. 4*F–I*
DP-378	2AFC	Male	C57BL/6J	8 w	12 w	Fig. 4*F–I*
DP-379	2AFC	Male	C57BL/6J	8 w	12 w	Figs. 4*C–I*, 5*A–D*; Movie 1; Extended Data Video 1
DP-380	2AFC	Male	C57BL/6J	8 w	12 w	Fig. 4*F–I*
DP-359	2AFC	Male	Rbp4-Cre	16 w	21 w	Fig. 5*F*,*G*

W, week.

#### Water restriction schedule

Water restriction was only applied on days with behavioral training/task and lasted a maximum of 12 consecutive days. More than 2 d with access to water *ad libitum* preceded every water restriction period. One-milliliter liquid (water and/or 10% sucrose water) per day was the target although reward (10% sucrose water) consumption during training/task could result in this being exceeded. If the animals consumed <1 ml reward during the behavioral training/task, water was supplemented as to reach the daily amount of liquid (i.e., a total of 1 ml). The animals’ health was carefully monitored, and their weight loss restricted to a maximum of 85% of their initial body weight.

### Surgical procedures

#### General procedures

The animals were deeply anesthetized with isoflurane in oxygen (4% for induction, 1–2% for maintenance), fixed in a stereotaxic frame (Harvard Apparatus), and buprenorphine (0.1 mg/kg, s.c.) was injected. Lidocaine (4 mg/kg, s.c.) was injected locally before skin incision. An ocular ointment (Bepanthen) was applied over the eyes, and the body temperature was maintained at 37°C using a heating pad. After surgery (details below), animals were injected with carprofen (5 mg/kg, s.c.) and returned to their homecage. An additional dose of carprofen was delivered 18–24 h after surgery.

#### Intracerebral virus injection

For virus injections, an incision into the skin overlying the skull was made and the skin was carefully moved aside. A small craniotomy (0.5 mm diameter) was drilled over the left orbitofrontal cortex (ORB; AP = 2.5 mm relative to bregma, DV = 1.6 mm relative to dura, ML = 1.0 mm relative to midline). Then, 400 nl virus (AAV1-CAG-FLEX-jGCaMP8m-WPRE; Addgene: 162381; titer: 2.2 × 10^13^) was delivered by a glass capillary attached to a motorized Quintessential Stereotaxic Injector (Stoelting) at a rate of 80 nl/min. The capillary was held in place for 5 min before the injection and for 10 min after the injection before being slowly retracted from the brain. The incision was closed with stitches (Ethicon).

#### GRIN lens and head-bar implantations

For head-bar implantation surgeries, the skin overlying the skull was removed and the bone was gently cleaned. A head-bar was attached to the exposed skull using a thin layer of super glue (Loctite) and covered using UV curable dental cement (Pluline).

Combined GRIN lens and head-bar implantation surgeries were performed 7 d after intracerebral virus injection. The existing craniotomy was enlarged (1.2 mm in diameter) and a 25 gauge needle was slowly lowered to 0.3 mm above the viral injection site for tunneling. The GRIN lens (G2P10, Thorlabs) was carefully lowered into the created tunnel to 0.2 mm above the viral injection site and held in place using UV curable dental cement. Subsequently, a head-bar was attached to the exposed skull as described above. The GRIN lens was protected with a custom plastic cover.

### Behavioral tasks and training schedules

Cohorts of mice (Table 1) were trained on auditory variants of behavioral tasks commonly used to study cognitive processes (Carandini and Churchland, 2013): a detection task (*n* = 3 mice), a Go/NoGo task (*n* = 4 mice), and a 2-alternative forced choice task (*n* = 4 mice). Behavioral experiments commenced Zeitgeber Time 4 to Zeitgeber Time 6.

#### Tone clouds

Tone clouds (adapted from Znamenskiy and Zador, 2013; Xiong et al., 2015; Marbach and Zador, 2017) were used as auditory cues in all three behavioral tasks (Fig. 1*B*). A tone cloud (100 tones/s) consisted of pure tones (30 ms duration) sampled from three different octaves [low (C7 to B7; 2,093–3,951 Hz), middle (C8 to B8; 4,186–7,902 Hz), or high (C9 to B9; 8,372–15,804 Hz)]. All octaves hold 12 different pitches/tones. The tones in a cloud were presented with 10 ms overlap, and each tone held rise and decay ramps (3 ms) to avoid on-/offset artifacts (The International Brain Laboratory et al., 2021).

The percentage value of a tone cloud, i.e., the stimulus strength, refers to the percent of tones that were sampled from one octave. For example, 100% tone clouds (used in the detection and Go/NoGo tasks as well as the early stages of the 2AFC task) consist of tones exclusively sampled from one octave, while in 70% tone clouds (used in the later stages in the 2AFC task), 70% of tones are sampled from either the low or the high octave, and 30% random tones are sampled from the other two octaves (equal probability of tones/octave). Tone clouds with lower stimulus strength are considered more difficult to perceive; conversely tone clouds with higher stimulus strength are considered easier to perceive. Tone clouds were sampled at 44,100 Hz and presented at 75–80 dB.

#### Liquid rewards

In all tasks, 10% sucrose water was used as liquid reward. To maximize the number of trials and to keep the animals motivated, intersession adjustment of the reward size was applied at certain training stages, i.e., the reward size in the current session was dictated by the amount of reward consumed in the previous session. If the animal had consumed >1 ml 10% sucrose water in total, the reward size was reduced by 0.1 µl compared with the session before, to a minimum volume (3 µl for the Detection and Go/NoGo tasks; 1.5 µl for the 2AFC task). Conversely, reward size was increased by 0.1 µl to a maximum volume (5 µl for the Detection and Go/NoGo tasks; 3 µl for the 2AFC task) if the animals consumed <1 ml 10% sucrose water on the prior training day.

#### Pretraining

All three tasks employed a pretraining stage (1 session/day for 3 consecutive days; session 1: 15 min, session 2: 30 min, session 3: 45 min) aimed to (1) habituate the animals to head fixation, (2) familiarize the animals to the auditory stimuli, and (3) teach the animals to consume reward from the licking spout. During the pretraining stage, the wheel was immobilized. Tone clouds (100%, 2.04 s duration) sampled from the middle (C8) octave were presented and reward thereafter delivered (5 µl 10% sucrose water). In all sessions, >200 tone clouds were presented.

#### Auditory detection task

**Figure 2.**
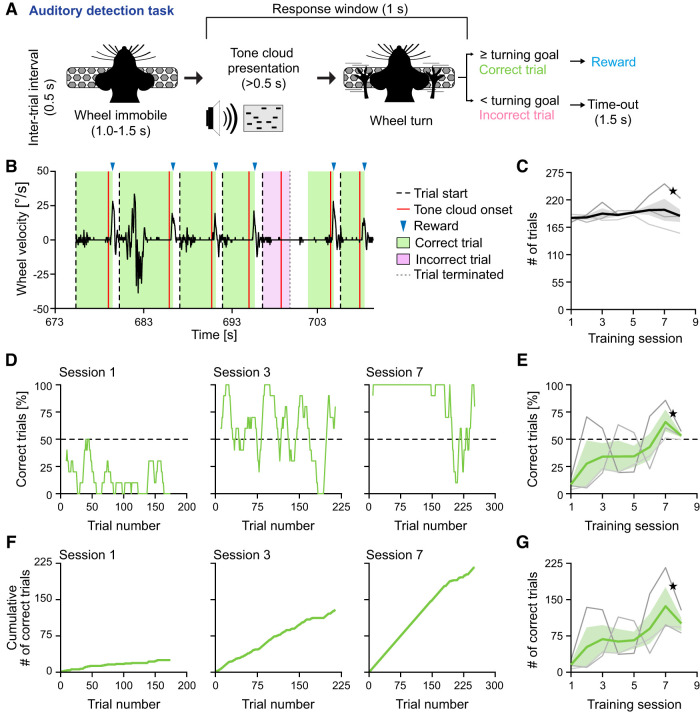
Implementing an auditory detection task using the DMC-Behavior Platform. ***A***, Schematic of the trial structure of the auditory detection task and the two different trial outcomes (correct or incorrect, respectively). ***B***, Example wheel turning behavior of a representative mouse during a random intrasession interval. The velocity (°/s) and the direction of the wheel turn (to right, positive values; to left, negative values) can be scored (black trace). Green shading, wheel turning ≥ turning goal = correct trial; pink shading, wheel turning < turning goal = incorrect trial. ***C***, Number (#) of trials per session during training, *n* = 3 mice. Black line, mean; gray shading, 68% CI; gray lines, individual mice. Black star, mouse in ***D***. ***D***, Performance of one mouse during individual training sessions (averaged over a rolling window of 10 trials). Correct trials, i.e., wheel turning ≥ turning goal are plotted. ***E***, Performance of *n* = 3 mice during the training. Correct trials, i.e., wheel turning ≥ turning goal are plotted. Green line, mean; green shading, 68% CI; gray lines, individual mice. Black star, mouse in ***D***. ***F***, ***G***, Same as ***D*** and ***E*** for cumulative count of correct trials.

After pretraining (see above, Pretraining), mice were trained to report (by turning the wheel to either right or left) the presentation of an auditory stimulus [100% tone clouds (0.51 s duration) sampled from the middle (C8 to B8) octave; Fig. 2*A*]. The wheel was movable throughout the training. Prior to each auditory stimulus presentation, the animal needed to refrain from turning the wheel for 1.0–1.5 s (allowing for 2 degrees jitter; see https://github.com/hejDMC/dmc-behavior for details). The auditory stimulus was presented and repeated until the animal responded (leading to immediate termination of stimulus presentation) or until the end of the response window (1.02 s from tone cloud onset). Intertrial intervals were 0.5 s. A session lasted ≥45 min and was terminated when the animal fulfilled a disengagement criterion (<4 wheel turns in the past 20 trials) or a time limit of 60 min was reached.

Two trial outcomes are possible: correct (wheel turn ≥ turning goal in response window) and incorrect (wheel turn < turning goal in response window). Correct trials resulted in reward delivery, while incorrect trials resulted in a 1.5 s time-out period.

Two training stages were used (stage 0 and stage 1):

Stage 0: a wheel turn of 15° (i.e., the turning goal) in response to stimulus presentation is rewarded with 5 µl 10% sucrose water. Animals advance to stage 1 after performing >150 correct trials in 1 session.

Stage 1: the turning goal is set to 30° (The International Brain Laboratory et al., 2021) and intersession adjustment of the reward size is applied (see above, Liquid rewards).

For data analysis, only trials within the first 20 min of a session were included.

#### Auditory Go/NoGo task

**Figure 3.**
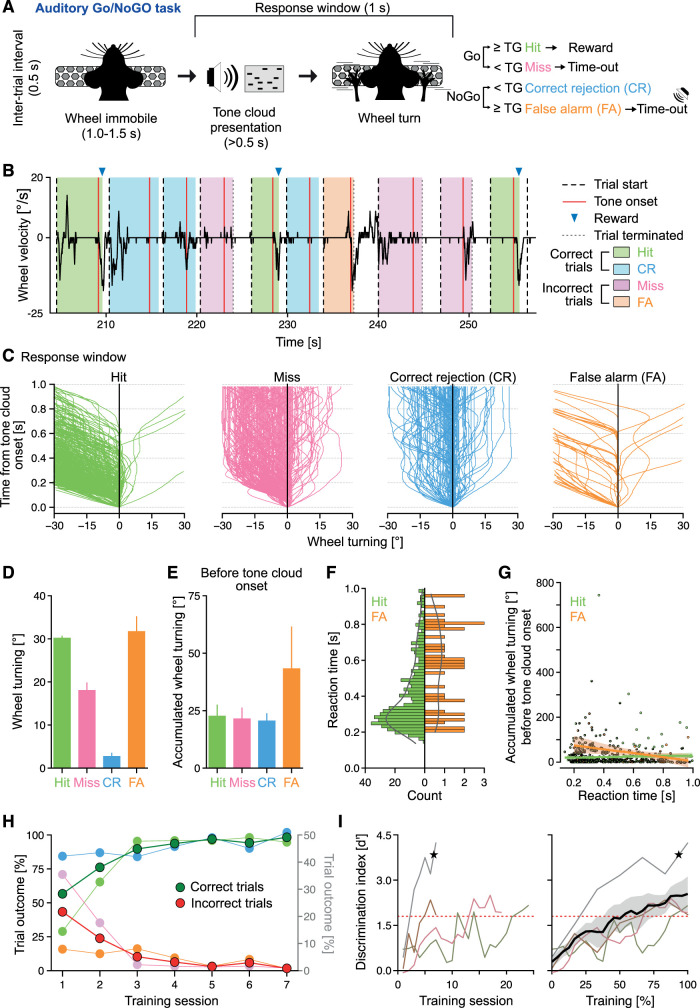
Implementing an auditory Go/NoGo task using the DMC-Behavior Platform. ***A***, Schematic of the trial structure of the auditory Go/NoGo task and the four different trial outcomes—Go trials: hit or miss, NoGo trials: correct rejection (CR) or false alarm (FA), respectively. ***B***, Example wheel turning behavior of a representative mouse during a random intrasession interval. The velocity (°/s) and the direction of the wheel turn (to right, positive values; to left, negative values) can be scored (black trace). Green shading, wheel turning ≥ turning goal in Go trails = hit; pink shading, wheel turning < turning goal in Go trails = miss; blue shading, wheel turning < turning goal in NoGo trails = correct rejection (CR); orange shading, wheel turning ≥ turning goal in NoGo trails = false alarm (FA). ***C***, The wheel turning (to left: negative values, to right: positive values) 0–1 s after tone cloud onset during the last three training sessions, same mouse as in ***B***. While this mouse prefers wheel turning to the left, the turning behavior differs between the four trial outcomes (*n*_hit_ = 514 trials, *n*_miss_ = 333 trials, *n*_CR_ = 832 trials, *n*_FA_ = 40 trials). One trace = one trial. ***D***, Summary of the maximum wheel turning in ***C***, mean ± SD, both turning directions included. ***E***, False alarm trials are associated with wheel turning before tone cloud onset. Accumulated wheel turning (mean ± SD, both turning directions) after trial start but before tone cloud onset (variable epoch lengths due to wheel immobility criteria, see Materials and Methods). Same animal as in ***B*** and same trials as in ***C***. ***F***, Distribution of the reaction time (i.e., time from tone cloud onset until the wheel turn exceeds the turning goal; RT) for hit trials (left panel, green) and false alarm (FA) trials (right panel, orange). Same data as in ***C***. ***G***, Accumulated wheel turning before tone cloud onset is negatively correlated with RT in false alarm (FA, orange), but not hit (green) trials. Scatter dots, individual trials; lines, linear regression fit; shaded area, 95% CI. ***H***, Trial outcomes across the training sessions for an example mouse. Left *y*-axis: cumulative percentage correct trials [dark green; hit trails + correct rejection (CR) trials] and incorrect trials [red; miss trials + false alarm (FA) trials], respectively. Right *y*-axis, percentage hit trials (light green), correct rejection (CR; blue), miss trials (pink), and false alarm (FA) trials (orange) across the training sessions. ***I***, Left, Behavioral performance (*d*’) of *n* = 4 mice across training sessions. Red dotted line, criterion for learning. Thin lines, individual mice; black star, mouse in ***H***. Right, Behavioral performance (*d*’) during training for the four mice in left, plotted based on time in training (%). Red dotted line, criterion for learning. Black line, mean; gray shading: 68% CI; thin lines, individual mice; black star, mouse in ***H***.

After pretraining (see above, Pretraining), mice were trained to turn the wheel in response to presentation of an auditory Go stimulus and to refrain from turning the wheel in response to presentation of an auditory NoGo stimulus. Then, 100% low (C7) and 100% high (C9) tone clouds (0.51 s duration; Fig. 3*A*), respectively, served as either Go or NoGo stimuli (randomly assigned to each animal at the beginning of training). The wheel was movable throughout the training. Prior to each auditory stimulus presentation, the animal needed to refrain from turning the wheel for 1.0–1.5 s (allowing for 2 degrees jitter; see https://github.com/hejDMC/dmc-behavior for details). The auditory stimulus was presented and repeated until the animal responded (leading to immediate termination of stimulus presentation) or until the end of the response window (1.02 s from tone cloud onset). Intertrial intervals were 0.5 s. Trial order (Go vs NoGo trials) was pseudorandom, restricting trial repeats to 3 (Banerjee et al., 2020). A session lasted ≥45 min and was terminated when the animal fulfilled a disengagement criterion (<4 wheel turns in the past 20 trials) or a 60 min time limit was reached.

Four trial outcomes are possible: hit (wheel turn ≥ turning goal in Go trial), false alarm (wheel turn ≥ turning goal in NoGo trial), correct rejection (wheel turn < turning goal in NoGo trial), and miss (wheel turn < turning goal in Go trial). Hits and correct rejections are counted as correct trials and false alarms and misses as incorrect trials. Hits resulted in reward delivery, while incorrect trials resulted in a 1.5 s time-out period. False alarms were additionally punished by a brief (0.5 s) white noise presentation. Correct rejections were neither rewarded nor punished. The animals’ performance was indicated by the discriminability index *d*’, calculated as the difference between the *z*-scored hit rate (*H*) and the *z*-scored false alarm rate (*F*) using the following formulas (Macmillan and Kaplan, 1985):

H=numberhits/(numberhits+numbermisses),

F=numberfalsealarms/(numberfalsealarms+numbercorrectrejections),

$$d^{\prime }=z(H)\;-\;z(F).$$

The training of animals consisted of two training stages (stage 0 and stage 1):

Stage 0: a wheel turn of 15° (i.e., the turning goal) in response to stimulus presentation is rewarded (Go trials) with 5 µl 10% sucrose water or punished (time-out and white noise; NoGo trials). Animals advance to stage 1 after performing >150 correct trials in 1 session.

Stage 1: the turning goal is set to 30° and intersession adjustments of the reward size are applied (see above, Liquid rewards). Task proficiency is assumed when the animals reach a *d*’ value of >1.8 on three consecutive sessions.

#### Auditory two-alternative forced choice (2AFC) task

**Figure 4.**
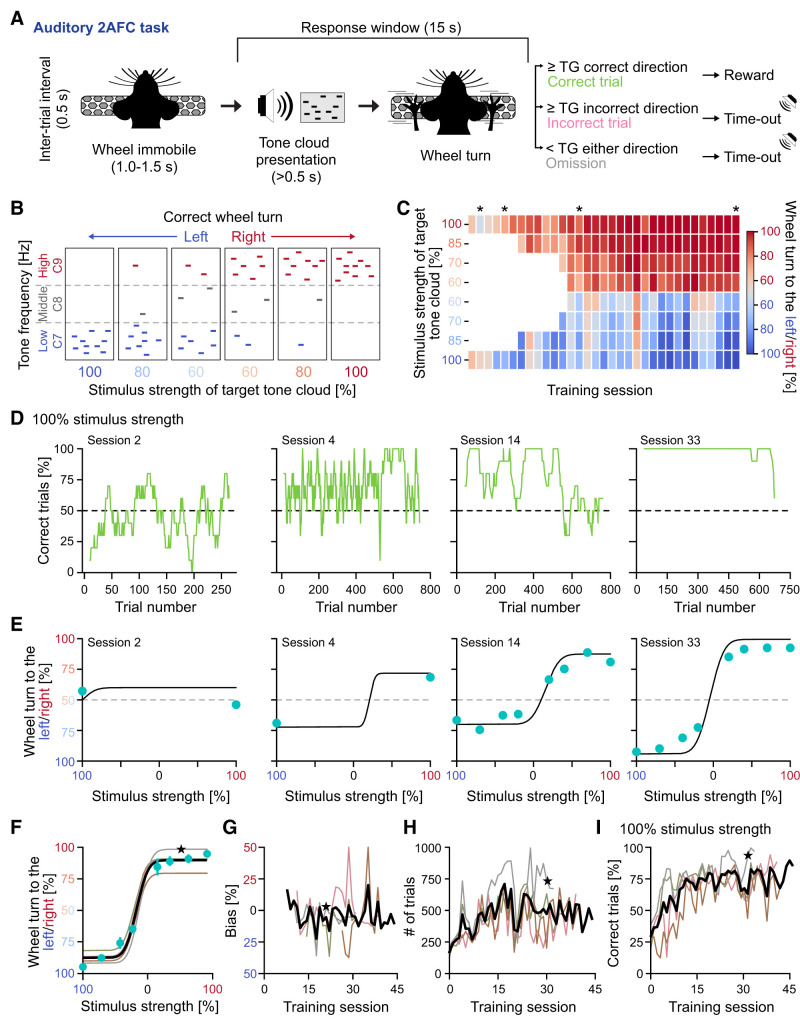
Implementing an auditory 2AFC task using the DMC-Behavior Platform. ***A***, Schematic of the trial structure of the auditory 2AFC task and different trial outcomes for correct/incorrect trials (wheel turns ≥ turning goal in correct/incorrect direction) and omissions (wheel turn < turning goal). ***B***, Schematic of the principle of tone clouds (black frames). Each stimulus strength is associated with a specific tone cloud composition, and the dominant octave (C7 or C9) dictates the correct wheel turn direction. ***C***, Wheel turning behavior across the training sessions of an example mouse, color-coded based on the percentage wheel turns to the right. Red colors, wheel turns predominantly to the right; blue colors, wheel turns predominantly to the left. The stimulus strength (%) dictates the correct wheel turn direction. One brick, one stimulus strength. This mouse occasionally turns the wheel to the right when the correct choice is to the left, but never the opposite. Black star, trials visualized in ***D*** and ***E***. ***D***, Performance (% correct trials) with tone clouds of 100% stimulus strength, i.e., 100% tone clouds, of one mouse during individual training sessions (averaged over a rolling window of 10 trials). Data from ***C***. ***E***, Psychometric curves for the sessions in ***D***. Turquoise dots, the fraction (%) wheel turns to the right (red) or to the left (blue) side as a function of the stimulus strength(s) used in the session. Black line, fitted psychometric curve. ***F–I***, Performance and bias during training, *n* = 4 mice. ***F***, Psychometric curves, the three last training sessions. Turquoise dots: the fraction (%) wheel turns to the right (red) or to the left (blue) side as a function of the stimulus strengths (mean 68% CI). Black line, mean; thin lines, individual mice. Black star, mouse in*** C–E***.*** G***, Bias in the wheel turning to the right (red) or to the left (blue). Bias was calculated for sessions that included 70% tone clouds to ensure accurate psychometric curve fitting (The International Brain Laboratory et al., 2021). Black line, mean; thin lines, individual mice. Black star, mouse in*** C–E***.*** H***, Number (#) of trials across the training sessions. Black line, mean, thin lines: individual mice; black star, mouse in ***C–E***. ***I***, The percentage correct trials, i.e., wheel turning ≥ turning goal to the correct direction in trials with 100% tone clouds. Black line, mean; thin lines, individual mice. Black star, mouse in ***C–E***.

After pretraining (see above, Pretraining), mice were trained to report tone clouds (0.51 s duration) of different difficulty levels by wheel turning in the correct direction—tone clouds with dominantly high tones (C9) should be reported with wheel turning in one direction and tone clouds with dominantly low tones (C7) with wheel turning in the other direction (the assignment of high/low tone clouds to left/right turns was performed randomly for each animal at the beginning of training; Fig. 4*A*). The wheel was movable throughout the training and prior to each auditory stimulus presentation the animal needed to refrain from turning the wheel for 1.0–1.5 s (allowing for 2 degrees jitter; see https://github.com/hejDMC/dmc-behavior for details). Throughout the training stages, tone clouds of different stimulus strength were proportionally presented across each session.

In each trial a tone cloud of a specific stimulus strength was presented and repeated until the animal responded (leading to immediate termination of stimulus presentation) or until the end of the response window (15 s from tone cloud onset). Intertrial intervals were 0.5 s. Inter- and intrasession debiasing was employed in stage 0–3. Intersession debiasing: if the animal displays a strong bias (>85% trials) in turning the wheel to either the left or the right side, the reward size was doubled for turning the wheel in the neglected direction on the subsequent training session. Intrasession debiasing: the trial order was pseudorandomized (The International Brain Laboratory et al., 2021) after incorrect trials (see https://github.com/hejDMC/dmc-behavior for details). A session lasted ≥45 min and was terminated when the animal fulfilled a disengagement criterion (rolling median of the reaction time over the last 20 trials was >4 times the session median reaction time) or a 90 min time limit was reached.

Three trial outcomes are possible: correct and incorrect trials (wheel turns ≥ turning goal in the correct/incorrect direction in response to stimulus presentation) and omissions (wheel turns < turning goal in response to stimulus presentation). Correct trials resulted in reward delivery (10% sucrose water), while incorrect trials and omissions resulted in a 1.5 s time-out period. Incorrect trials were additionally punished by a brief (0.5 s) white noise presentation.

The training of animals consisted of five training stages (stage 0 to stage 4; modified from Appendix 2 of The International Brain Laboratory et al., 2021):

Stage 0: 100% tone clouds are used, and the turning goal is 15°. High (C9)/low (C7) tone clouds are presented in alternating blocks (tone cloud presentation was repeated *n* trials until the animal performed three subsequent correct trials). Animals advance to stage 1 after performing >300 correct trials in 1 session.

Stage 1: 100% tone clouds are used, and the turning goal is 30°. High (C9)/low (C7) tone clouds are pseudorandomly presented. Animals advance to stage 2 after performing >80% correct trials for both high and low tone cloud presentations in 1 session.

Stage 2: 100 and 85% tone clouds are used, and the turning goal is 30°. Animals advance to stage 3 after performing >75% correct trials for both high and low tone cloud presentations in 1 session.

Stage 3: 100, 85, and 70% tone clouds are used, and the turning goal is 30°. Animals advance to stage 4 after performing >350 correct trials in 1 session.

Stage 4: 100, 85, 70, and 60% tone clouds are used, and the turning goal is 30°. Task proficiency is assumed when a set of criteria is fulfilled in stage 4: (1) the animal performs >300 trials on three subsequent sessions, respectively; (2) the animal performs >80% correct trials on both 100% high and low tone clouds on three subsequent sessions, respectively; and (3) the psychometric curve (The International Brain Laboratory et al., 2021) calculated for the cumulative training data on these three sessions displays a bias of <16%, threshold of <19% and lapse rates of <0.2. Psychometric curves were fitted using an error function (erf) of the form:

$$P(x)=\mathrm{laps}e_{1}+(1-\mathrm{laps}e_{1}-\mathrm{laps}e_{2})\;*\;\left(\frac{\mathrm{erf}\left(\frac{x-\mathrm{bias}}{\mathrm{slope}}\right)+1}{2}\right).$$

The parameters were estimated using maximum likelihood estimation (MLE, The International Brain Laboratory et al., 2021).

### 2-Photon calcium imaging and analysis

2-Photon calcium imaging data were acquired at 31 frames per second (fps; 512 × 512 pixels, 600 × 600 μm FOV) using a custom-built two-photon microscope (INSS) equipped with an 16× objective (N16XLWD-PF, Nikon) using the SciScan acquisition software. The excitation wavelength was 950 nm using an InSight X3 laser (Spectra-Physics). The average power measured under the objective was ∼100 mW. Photons were detected using GaAsP photomultiplier tubes (PMT2101/M, Thorlabs). Frame pulses were recorded with the Raspberry Pi and synchronized to the behavioral data post hoc. For processing of the imaging data (motion correction, ROI detection, cell classification, neuropil correction), the *suite2p* toolbox (Pachitariu et al., 2017) was used. The calcium traces are presented as *dF/F* values.

### DeepLabCut analysis

To reconstruct forepaw movements and licking, videos of the animals were recorded at 30 Hz using a Blackfly S USB3 camera (Teledyne FLIR). Video frames were triggered by the Raspberry Pi and synchronized to the behavioral data post hoc. We used DeepLabCut (DLC; Mathis et al., 2018) for tracking of both forepaws and the tongue. Extracted trajectories were analyzed and visualized using Python.

### Data analysis and figure design

**Figure 5.**
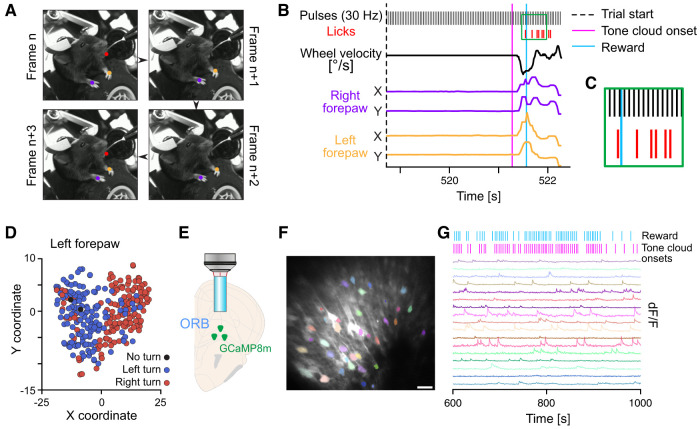
Integration of external recording devices with the DMC-Behavior Platform. ***A–D***, The DMC-Behavior Platform was used to trigger continuous camera frames to record forepaw movement and licking behavior of a mouse performing the 2AFC task. ***A***, Individual camera frames triggered by TTL pulses from the DMC-Behavior Platform. Dots mark DLC-tracked poses of the left (orange) and the right (lilac) forepaws and of the tongue (red). ***B***, Example of synchronized data. Dashed black line, trial start; pink line, tone cloud onset; blue line, reward delivery. Black trace, wheel velocity; lilac and orange traces, *x*, *y* positions of right and left forepaw, respectively. ***C***, Green frame in ***B***. Black bars, pulses (30 Hz) for triggering camera frames; red bars, licks; blue line, reward delivery. ***D***, t-SNE clustering and visualization of the *x*, *y* position of the left forepaw 0–1 s after tone cloud onset. One dot, one trial; colors reflect the wheel turning behavior. ***E–G***, Synchronization of calcium imaging with behavior in the 2AFC task using the DMC-Behavior Platform. ***E***, Schematic outline of the experimental design. GCaMP8m was virally targeted to the ORB in Rbp4-Cre mice and imaged through a GRIN lens using a 2-photon microscope. ***F***, Maximum intensity projection of individual ORB neurons expressing GCaMP8m (colored masks used for extraction of calcium signals). Scale bar, 50 μm. ***G***, Calcium transients of the ORB neurons in ***F*** and the temporal relationship to tone cloud onset and reward delivery controlled by the DMC-Behavior Platform*.* Pink bars, tone cloud onset; blue bars, reward delivery.

All data was analyzed and visualized using Python3.9 and the following packages: matplotlib (3.7.1), natsort (8.4.0), numpy (1.24.2), opencv-python (4.9.0.80), pandas (1.5.3), scipy (1.10.1), seaborn (0.12.2), and statsmodels (0.14.1). Schematics for the mouse (graphical abstract) and the mouse head (Figs. 2*A*, 3*A*, 4*A*) were adapted from scidraw.io. Schematics for brain sections (Fig. 5*D*) were created and adapted from the DMC-BrainMap pipeline (Jung et al., 2025; https://github.com/hejDMC/napari-dmc-brainmap). All figures were assembled in Inkscape and Adobe Illustrator. If not otherwise stated, data is presented as mean ± standard deviation. Statistical significance was assumed for *p* values <0.05. For correlation analysis, Pearson’s correlation coefficients were computed, the Friedman test was performed to compare data across training sessions and linear regression models were fitted using the ordinary least squares method.

## Results

### Auditory detection task

As a first step, we used the DMC-Behavior Platform to implement a simple auditory detection task for establishing the wheel-based approach for assaying auditory driven decision-making. A cohort of mice (*n* = 3) underwent training to report the presence of a tone cloud (C8; Materials and Methods) by turning the wheel to either direction (Fig. 2*A*). In this simple form, the auditory detection task can be interpreted as a variant of operant conditioning (Thorndike, 1898; 1933) that can be used to, e.g., investigate the neuronal underpinnings of basic, auditory reinforcement learning.

To document the decision-making behavior in response to auditory stimulus presentation, the wheel turning behavior was recorded with high spatial [1,024 pulses per revolution (PPR)] and temporal resolution (100 Hz). Fulfillment of the turning goal (wheel turning >30°) during the response window resulted in reward delivery (Fig. 2*B*). The number of trials performed did not differ across training sessions and stages (Friedman Test; *X*^2^_(7)_ = 3.39, *p* = 0.850) yielding on average 188.0 ± 28.0 trials (i.e., correct and incorrect trials; Fig. 2*C*).

The animals’ performance of the task was estimated by two, related metrics: the proportion of correct trials (Fig. 2*D*,*E*) and the number of correct trials (Fig. 2*F*,*G*), revealing gradually improved performance over the course of the training sessions (*r*_%correct(6)_ = 0.91, *p* = 0.002; *r*_#correct(6)_ = 0.88, *p* = 0.004), albeit notable intrasession variability of the animals’ performance was observed (Fig. 2*D*,*F*). In conclusion, our results underscore the utility of the wheel-based approach for assaying auditory driven decision-making as employed by the DMC-Behavior Platform.

### Auditory Go/NoGo task

Next, we used the DMC-Behavior Platform to implement a staged training protocol of an auditory Go/NoGo task. Go/NoGo tasks are commonly used for probing perceptual decision-making (Carandini and Churchland, 2013), motivation (Berditchevskaia et al., 2016; Matteucci et al., 2022), impulsivity (Berditchevskaia et al., 2016; Li et al., 2020), attention, and short-term memory (Kamigaki and Dan, 2017) and rule switching behaviors (Banerjee et al., 2020). Here, head-fixed mice (*n* = 4) were trained to either turn (Go trial) or refrain from turning (NoGo trial) the steering wheel in response to distinct auditory stimuli composed of tone clouds (Materials and Methods and Fig. 3*A*). As general to Go/NoGo tasks, four behavioral outcomes were possible: hit (wheel turn ≥ turning goal in Go trial), false alarm (wheel turn ≥ turning goal in NoGo trial), correct rejection (wheel turn < turning goal in NoGo trial), and miss (wheel turn < turning goal in Go trial; Fig. 3*A*). All mice fulfilled the criterion to advance to training stage 1 after 1 session in training stage 0. The animals performed a high number of trials (536.8 ± 36.0) during training (13.0 ± 7.6 sessions) to reach task proficiency. The steering wheel approach employs wheel turning goals as criteria for the animals’ behavioral responses, and a continuous measure (degrees of wheel turning) is thus used for read-out of the animal's choice. Continuous measures can provide insight into latent components underlying the behavior (Burgess et al., 2017) that are difficult to approach with categorical response measures (e.g., licks). Capitalizing on this, we analyzed the wheel turning for one mouse in detail (last three sessions combined: *n*_hit_ = 514 trials, *n*_miss_ = 333 trials, *n*_CR_ = 832 trials, *n*_FA_ = 40 trials). The wheel turning's structure, velocity, and timing are metrics that can be analyzed. Interestingly, the analyses showed that wheel turning was executed in all four trial outcomes, including miss trials and correct rejection trials (Fig. 3*B–D*). While hit trials and false alarm trials were associated with the highest degree of wheel turning, false alarm trials were uniquely signified by extensive wheel turning (43.4 ± 53.1°) before tone cloud onset, possibly reflecting an impulsive state (Fig. 3*D*,*E*). Reaction time (RT) is one of the most widely utilized metrics to infer content and duration of mental operations and is measured by the time elapsed between stimulus onset and the organism's response, in our task defined as the time between tone cloud onset and reaching of the turning goal. The RT is thus only calculable for hit and false alarm trials, respectively. While hit trials were characterized by fast RT, the RT for false alarm trials varied (Fig. 3*F*). We reasoned that if the prominent wheel turning before tone cloud onset in false alarm trials was a reflection of an impulsive state, this state should also impact the RT, potentially explaining why the RT varied across false alarm trials. Indeed, we identified a significant negative correlation between the accumulated wheel turning before tone cloud onset and the RT in false alarm trials but not in hit trials (FA: *R*^2^ = 0.143, *F*_(1,38)_ = 6.353, *p* = 0.016; Hit: *R*^2^ = 0.002, *F*_(1,512)_ = 0.8451, *p* = 0.36; Fig. 3*G*). In essence, the more wheel turning before the tone cloud onset, the faster the turning goal was reached in false alarm trials, a correlation not found in hit trials.

Over the course of training, we found a gradual increase in performance resulting in effective discrimination of high and low tone clouds serving as Go/NoGo stimuli (Fig. 3*H*,*I*; *d*’ = 2.46 ± 0.87 on the last training session vs *d*’ = 0.29 ± 0.35 on the first training session). Interestingly, two mice learned the task very fast (6.0 ± 1.0 sessions) while the other two required significantly more training (20.0 ± 4.0 sessions; Fig. 3*I*).

### Auditory two-alternative forced choice (2AFC) task

Go/NoGo tasks demand subjects to inhibit behavioral output in response to specific stimuli (NoGo trials). Hidden factors such as impulsivity thus constitute a potential confound (Carandini and Churchland, 2013). Furthermore, as a rule, Go/NoGo tasks employ asymmetric trial outcomes, with one type of correct trials being rewarded (hit) and another type of correct trials not being rewarded (correct rejection). The different types of errors (miss vs false alarm) can also entail differential outcomes, as in our study. 2AFC tasks, on the contrary, do not entail response inhibition but instead force the subject to choose one of two options in response to different sensory stimuli. 2AFC tasks facilitate the isolation of factors involved in perceptual decision-making, including sensory information, arousal, motivation, or motor biases (Carandini and Churchland, 2013). We used the DMC-Behavior Platform to implement an auditory 2AFC task asking the mice to turn the steering wheel in a certain direction (left or right) in response to presentation of specific tone clouds (Fig. 4*A*,*B*). Over the course of training perceptually more difficult tone clouds were successively introduced, i.e., the discriminability between high and low tone clouds decreased (Materials and Methods and Fig. 4*C*). The performance on trials using 100% tone clouds gradually increased during training, illustrating effective discrimination between different high/low tone clouds (Fig. 4*D*), while, as expected, the performance in trials using perceptually more difficult tone clouds was comparably lower (Fig. 4*C*).

We used the behavioral data provided by DMC-Behavior Platform to calculate high-quality psychometric curves (Fig. 4*E*,*F*), a method commonly employed to relate cognition, perception, and behavior to understand how (noisy) sensory information gives rise to decisions (Carandini and Churchland, 2013). The asymptotes of the psychometric curves allow the estimation of the lapse rates, i.e., the errors mice made in trials with easy to perceive stimuli (100% trials; Materials and Methods). We found low lapse rates to the left (0.13 ± 0.04) and right side (0.10 ± 0.07), respectively (Fig. 4*F*), indicating that mice were able to effectively discriminate easy to perceive stimuli. Horizontal displacement of the psychometric curve indicates a bias in the wheel turning (to the right or to the left). We observed that mice could display highly biased wheel turning during specific training sessions (Fig. 4*C*), but upon task proficiency only a minor bias remained (toward left wheel turns; 6.89% ± 1.59%; Fig. 4*G*).

The training design (see Materials and Methods) encouraged the mice to perform a large number of trials (18,471.75 ± 1,834.78 trials, *n* = 4 mice) across training sessions (525.34 ± 113.82 trials/session; Fig. 4*H*), thus promoting the effective learning of the task (Fig. 4*I*). Overall, this line of investigation demonstrates the utility of the DMC-Behavior Platform for training animals in complex, auditory-driven 2AFC tasks paralleling established visual paradigms (The International Brain Laboratory et al., 2021).

### Synchronization with external recording devices

After establishing auditory decision-making tasks of varying complexity levels, we confirmed the integration of the DMC-Behavior Platform with external devices connected to the GPIO pins of the Raspberry Pi (Fig. 1*B*). In a first set of experiments, we used the platform to generate and send synchronization signals (a continuous 30 Hz TTL signal) to a camera, triggering individual frame images of the face and forepaws of a mouse engaged in the auditory 2AFC task (Fig. 5*A*). We observed interframe intervals of 34.0 ± 1.2 ms and no dropped frames. To temporally reconstruct the animals’ reward-licking and wheel turning, the position of the tongue (Movie 1; Extended Data Video 1) and of the forepaws, respectively, was extracted and aligned to relevant task events (Fig. 5*B*,*C*). The analysis of the forepaw position indicated the emergence of two distinct clusters, putatively representing left and right wheel turns, respectively (Fig. 5*D*). Integration of the forepaw position revealed by DLC with the behavioral data registered by the DMC-Behavior Platform (left vs right turns) revealed an almost perfect match, indicating successful integration of the external camera with the DMC-Behavior Platform.

In a second set of experiments, 2-photon calcium imaging of neuronal activity was included in an animal conducting the auditory 2AFC task (Fig. 4). The activity of a population of excitatory neurons in the orbitofrontal cortex (ORB) was imaged through a GRIN lens at a frame rate of 31 Hz (Fig. 5*E*). Frame pulses from the 2-photon microscope were recorded by the DMC-Behavior Platform and stored offline (no dropped frames; inter-frame interval: 32.3 ± 1.6 ms; see extended discussion in/tutorials/synchronization_test in Extended Data 1). This allowed for successful synchronization of extracted calcium transients from individual neurons to task events, e.g., tone cloud onsets, and reward deliveries (Fig. 5*F*,*G*). Hence, future work combining the DMC-Behavior Platform with 2-photon calcium imaging could yield valuable insights into the neural correlates of decision-making in auditory tasks.

## Discussion

Standardization is key to reproducibility of measurements of mouse behavior across studies and laboratories (Kafkafi et al., 2018; The International Brain Laboratory et al., 2021) and hence critical to scientific progress in (systems) neuroscience. We here introduce the DMC-Behavior Platform, a platform for standardized and replicable study of decision-making in mice. The platform is built on hardware components established by the IBL consortium (Abbott et al., 2017), particularly the utilization of a steering wheel to record the behavioral responses of the animal (Burgess et al., 2017; The International Brain Laboratory et al., 2021, see also: Aoki et al., 2017; Bloem et al., 2022). This, and our implementation of a probabilistic auditory 2AFC task analogously to the task version used by the IBL consortium (The International Brain Laboratory et al., 2021), offers comparative studies between visual and auditory decision-making. Importantly, the steering wheel approach, in contrast to licking-based approaches, dissociates the encoding of reward consumption (licking) from motor commands expressing an animal's decision (turning the wheel), arguably facilitating decoding of decision-making variables in neuronal circuits. As discussed previously (Burgess et al., 2017), the continuous read-out of the wheel position offers insights into e.g., the motivational state or confidence of the animal. As a proof of concept, we show that wheel turning activity before tone cloud onset—a putative measure of the animal's impulsivity—can be related to the reaction time, with higher wheel turning before tone cloud onset negatively correlating with reaction time in false alarm trials but not hit trials (Go/NoGo task; Fig. 3*G*). However, a downside of using a steering wheel is that the mice are trained on ethologically unrealistic behaviors, likely increasing the training duration (Marbach and Zador, 2017).

A general limitation of current platforms used to study (auditory) decision-making in head-fixed mice is their restriction to a single defined behavioral task (Sanders and Kepecs, 2012; Marbach and Zador, 2017). The DMC-Behavior Platform aims at overcoming this limitation by allowing users to flexibly design and customize existing tasks, extensions that can easily be shared online for other laboratories to replicate. We, in the present study, exemplify this concept by implementing auditory variants of three distinct, commonly used decision-making paradigms.

One critical factor that often increases variability in behavioral data is the experimenter themselves (Kafkafi et al., 2018). As more complex tasks often require staged training procedures, manual adjustments between—or even within—sessions are often a necessity, adaptations that are error prone. The variability risks to be further increased if several experimenters conduct the work. The DMC-Behavior Platform minimizes the experimenters’ impact on data variability by fully automatizing procedures from training initiation to task execution. This includes automated monitoring of stage progression, optimization of intra-/intersession factors (reward size adjustments, debiasing protocols) and data storage (see https://github.com/hejDMC/dmc-behavior for details). A beneficial side effect is reduced workload for the experimenter.

A limitation of Raspberry Pis is that, e.g., system overhead, interrupt handling, thread scheduling, and garbage collection increase the timing variability of CPU-bound operations such as triggering/recording of time-series data (e.g., camera/2-photon frame pulses). We, however, observed that timing variability occurs at behaviorally irrelevant timescales (low, single-digit ms range), without significant effect on the alignment of time-series to behavioral data (see extended discussion in/tutorials/synchronization_test in Extended Data 1). Nonetheless, the DMC-Behavior Platform is not suitable for implementing behavioral tasks requiring submillisecond precision or recording of frame pulses/timestamps at high sampling rates (>1 kHz). Instead, we recommend using the platform to generate a (slower) synchronization signal (20–60 Hz) to be concurrently recorded alongside the time-series data (e.g., fiber photometry or electrophysiology). The synchronization signal can be used for post hoc alignment of time-series to behavioral data (see data presented in Fig. 5*A–D*).

In summary, the DMC-Behavior Platform allows for seamless integration of behavioral data acquisition with external devices to record or manipulate neuronal/behavioral activity, providing a variety of opportunities to test novel as well as long standing hypotheses on the neuronal underpinnings of cognitive processes utilizing auditory information.

## Synthesis

Reviewing Editor: Mark Laubach, American University Decisions are customarily a result of the Reviewing Editor and the peer reviewers coming together and discussing their recommendations until a consensus is reached. When revisions are invited, a fact-based synthesis statement explaining their decision and outlining what is needed to prepare a revision will be listed below. The following reviewer(s) agreed to reveal their identity: NONE.

Thanks for sending your work to eNeuro. Your paper was reviewed by two experts. Their comments are below. Please revise your manuscript to address all points that were raised.

Reviewer 1

Advances the Field

The authors present DMC-Behavior, an open-source method for training head-fixed mice on decision-making tasks using auditory stimuli. The tool can be built from generally available and inexpensive components and applied to a variety of operant conditioning tasks. Several such tasks are included with an organized, coherent code base publicly available on GitHub. Care has been taken to provide options to synchronize with a variety of components such as cameras or other neuroscience recording equipment. The authors include a convincing demonstration of the utility of their method by means of an auditory discrimination test that yielded a high-quality, multifaceted dataset indicating expected results. While individual features of this method are not unique, the package would be useful for laboratories that conduct such head-fixed auditory conditioning experiments and especially for laboratories for which the extensible nature of the project can be leveraged for a variety of purposes.

Statistics The model fit for psychometric curves isn't stated (Gaussian?).

Visual Abstract

Suggest using Figure 1 or a modified version for the visual abstract.

Software

I do not have access to the hardware required, but I did look over the code base. The startup instructions appear clear, comprehensive, and organized.

Constructive feedback:

1. Moving hard-coded values to the start of the file a la PEP8 would makes the code more easily readable and modifiable (e.g., rotation_value = 1024/360, HIGH = 'high'). Even better, they might be moved to a settings file.

2. If, as per line 463, the authors imagine users flexibly designing or modifying tasks, some thought might be given to breaking out methods likely to be shared between many experiment types (such as logging data, playing tones, etc.) from the very large classes that run specific experiment types into helper functions or classes that might be used by users to more easily modify or construct those new tasks.

3. System clocks on Raspberry Pis have variance in the single-digit millisecond range. This isn't particularly relevant for the 30 fps camera/2p, but it would matter if a user needed to actually leverage the default report rate of 1kHz or higher. Using an inexpensive RTC module would remove this limitation and increase the flexibility to be used for applications such as fiber photometry.

Comments to the Authors

Shapeways is listed as a vendor for some parts (and one part is 'custom'). It would be helpful for any potential adopters to provide any 3-D print designs in the GitHub repo so they might be able to lower costs by printing their own parts.

Similarly, a sample of what data output should look like would help prospective users troubleshoot results or design their analysis pipelines. The level of organization of data output is a big factor in whether or not I find a tool useful!

Line 139-140: Super Glue is not a proper noun and can be in lower case.

Line 154: The nested parenthetical may be omitted.

Line 182: The numeral form is generally preferred (e.g., 1 session/day)

Line 213: The % signs should follow the number without a space.

Line 315: This link 404s: https://github.com/hejDMC/napari-dmc-brainmap

Figure 4: There is a typo throughout ('strenght')

Reviewer 2

Major comments:

My main concern is the capability of the Raspberry pi to precisely record and control multiple signals simultaneously (the wheel signals, cameras triggering, 2hv camera signal). Indeed, in contrast to single-core devices (such as Arduino or teensy), which operate on a single clock, the Raspberry pi has multiple cores. While this is very interesting for creating complex code or performing higher level of computation, it is generally not recommended for real-time tasks. Indeed, the module RPi.GPIO, used in some of the submitted codes (e.g. sync_pulse.py), seems "[...] unsuitable for real-time or timing critical applications", as described in the library documentation (https://pypi.org/project/RPi.GPIO/). To some extent, this limitation explains why Arduino board are widely used for open-source projects (e.g. PulsePal). Therefore, I would ask the authors to comment and discuss this critical point.

Additionally, the authors may be able to calculate the Raspberry pi 'jitter' for the recorded and sent signals of their setup. By simultaneously recording several signals with a classical DAC (e.g. oscilloscope, national instrument DAC, openEphys or CED) versus a Raspberry, should be feasible and fast to realize such a measure. In my opinion, the interesting t-SNE clustering (figure 5) does not provide a direct proof of the camera synchronization accuracy. However, it suggests that at slow sampling rate, like the one used in this manuscript (100 Hz), synchronization is probably not dramatically affected. Testing different sampling rates could provide further insights and be used as an argument to strengthen the manuscript.

I would like to emphasize the quality of the manuscript and recommend its publication following the authors' responses to my major comment. I am confident that this manuscript will help researchers working on behavioural experiments customize their methods to fulfill their experimental needs.

Minor comments:

- Line 51: To complete the reference list ("Steinmetz et al., 2021; Zong et al., 2022"), I suggest adding a reference that use large-scale 1photon-miniscope imaging.

- Line 152: Since it is not very common in behavioral training to use ZT4 to ZT6, I would describe the abbreviation 'Zeitgeber Time'.

-

- Line 181: I suggest changing the title to 'Pretraining.' During this phase, the mice associate the cue with the reward. In my opinion, it is a Pavlovian conditioning rather than habituation.

-

- Line 190, 208, 211: The authors use the expression "see above". To assist the reader, consider referring to the specific section title in the manuscript.

-

- Line 233. Could you please describe the abbreviation '#' ' for clarity?

## Author Response

Response to reviewers --------------------------------------------- Synthesis of Reviews:

Significance Statement Comments for Author (Required):

N/A Comments on the Visual Abstract for Author (Required):

Please use Figure 1 or a modified version of it for this component of the manuscript submission.

Synthesis Statement for Author (Required):

Thanks for sending your work to eNeuro. Your paper was reviewed by two experts. Their comments are below. Please revise your manuscript to address all points that were raised.

Reviewer 1 Advances the Field The authors present DMC-Behavior, an open-source method for training head-fixed mice on decision-making tasks using auditory stimuli. The tool can be built from generally available and inexpensive components and applied to a variety of operant conditioning tasks. Several such tasks are included with an organized, coherent code base publicly available on GitHub. Care has been taken to provide options to synchronize with a variety of components such as cameras or other neuroscience recording equipment. The authors include a convincing demonstration of the utility of their method by means of an auditory discrimination test that yielded a high-quality, multifaceted dataset indicating expected results. While individual features of this method are not unique, the package would be useful for laboratories that conduct such head-fixed auditory conditioning experiments and especially for laboratories for which the extensible nature of the project can be leveraged for a variety of purposes.

Statistics The model fit for psychometric curves isn't stated (Gaussian?).

We thank the reviewer for identifying this omission, we have added the missing information (line 296): "Psychometric curves were fitted using an error function (erf) of the form:

P(x)= 〖lapse〗_1+(1- 〖lapse〗_1- 〖lapse〗_2 )*((erf⁡(((x-bias))⁄slope)+1)/2) The parameters were estimated using maximum likelihood estimation (MLE, Aguillon-Rodriguez et al., 2021)." Visual Abstract Suggest using Figure 1 or a modified version for the visual abstract.

We thank the reviewer for this suggestion. We have added a visual abstract based on Figure 1.

Software I do not have access to the hardware required, but I did look over the code base. The startup instructions appear clear, comprehensive, and organized.

We appreciate the reviewer's positive feedback.

Constructive feedback:

1. Moving hard-coded values to the start of the file a la PEP8 would makes the code more easily readable and modifiable (e.g., rotation_value = 1024/360, HIGH = 'high'). Even better, they might be moved to a settings file.

2. If, as per line 463, the authors imagine users flexibly designing or modifying tasks, some thought might be given to breaking out methods likely to be shared between many experiment types (such as logging data, playing tones, etc.) from the very large classes that run specific experiment types into helper functions or classes that might be used by users to more easily modify or construct those new tasks.

Regarding comments 1 and 2: We sincerely appreciate the reviewer's constructive feedback. In response, we extensively refactored our code base. Specifically, we formatted all code according to the PEP8 standard and introduced a base task class to consolidate shared functionalities across individual tasks. Additionally, we created dedicated 'manager' modules to handle common processes such as data logging, reward delivery, and stimulus management. Lastly, we streamlined the user experience by consolidating habituation/pretraining and training scripts into single files, eliminating the need to run individual files for different tasks. We believe these improvements will significantly enhance usability and facilitate task construction and modification for users.

3. System clocks on Raspberry Pis have variance in the single-digit millisecond range. This isn't particularly relevant for the 30 fps camera/2p, but it would matter if a user needed to actually leverage the default report rate of 1kHz or higher. Using an inexpensive RTC module would remove this limitation and increase the flexibility to be used for applications such as fiber photometry.

We thank the reviewer for this thoughtful comment. Indeed, timestamping continuous input/output variables (e.g. the frame pulses of the camera/2p) with a Raspberry Pi will result in some variance of the inter-timestamp intervals. We observed an inter-timestamp variance of 1.37 * 10-3 ms2 for the camera (data from Figure 5A-D) and 5.86 * 10-4 ms2 for the concurrent rotary encoder data. For the 2p frame pulses (data from Figure 5E-G), we found a variance of 0.25 ms2 for the frame pulses and 6.01 * 10-4 ms2 for the concurrent rotary encoder data. We agree with the reviewer that this variation is neglectable for the imaging experiments in the present study.

We agree with the reviewer that the DMC-Behavior Platform is not optimally suited to record frame pulses at higher (> 1 kHz) sampling rates from e.g. fiber photometry experiments. A common practice for synchronizing behavioral to continuously recorded input/output data with high sampling rates (e.g. fiber photometry or electrophysiological recordings) is to generate a (slower) synchronization signal (at 20 - 60 Hz) that is used for post-hoc alignment of the high-sampling rate dataset to the behavioral data. We demonstrate in Figure 5A-D the capability of the DMC-Behavior Platform to do just that. For making this explicit, we added the following sentences to the Discussion (line 516): "A limitation of Raspberry Pis is that e.g. system overhead, interrupt handling, thread scheduling and garbage collection increase the timing variability of CPU-bound operations such as triggering/recording of time-series data (e.g. camera/2-photon frame pulses). We, however, observed that timing variability occurs at behaviorally irrelevant timescales (low, single-digit ms range), without significant effect on the alignment of time-series to behavioral data. Nonetheless, the DMC-Behavior Platform is not suitable for implementing behavioral tasks requiring sub-millisecond precision or recording of frame pulses/timestamps at high sampling rates (> 1 kHz). Instead, we recommend using the platform to generate a (slower) synchronization signal (20-60 Hz) to be concurrently recorded alongside the time-series data (e.g. fiber photometry or electrophysiology). The synchronization signal can be used for post-hoc alignment of time-series to behavioral data (see data presented in Figure 5A-D)." We sincerely thank the reviewer for the advice on using an RTC module to enhance the recording/timestamping of high frequency time-series data. Modules such as the DS3231 and DS1307, which provide programmable square wave signals up to 32 kHz, offer an accurate clock source. However, retrieving time information from an RTC module via the I2C interface occurs at much slower rates (1 Hz). At this point, we believe that our current approach-generating a slower synchronization signal (20 - 60 Hz) for post-hoc alignment-provides an effective and practical solution. Nevertheless, we plan to explore enhancements, including the incorporation of an RTC module, in future iterations of the DMC-Behavior Platform.

Comments to the Authors Shapeways is listed as a vendor for some parts (and one part is 'custom'). It would be helpful for any potential adopters to provide any 3-D print designs in the GitHub repo so they might be able to lower costs by printing their own parts.

We thank the reviewer for this helpful comment. We have added the .stl file of the speaker mount labelled as 'custom' to the GitHub repository. The .stl files for the other 3D-printable objects are adapted from the IBL consortium and can be found via the links provided in the parts list file.

Similarly, a sample of what data output should look like would help prospective users troubleshoot results or design their analysis pipelines. The level of organization of data output is a big factor in whether or not I find a tool useful! We appreciate the reviewers constructive feedback and added some example data to the GitHub repository (./tutorial). Alongside, we include scripts for data analysis.

Line 139-140: Super Glue is not a proper noun and can be in lower case.

We thank the reviewer for catching this error, and the spelling has been corrected.

Line 154: The nested parenthetical may be omitted.

We thank the reviewer for pointing this out, we now omit nested parentheticals in lines 156, 277 and 315 .

Line 182: The numeral form is generally preferred (e.g., 1 session/day) We appreciate the reviewers feedback and have corrected the spelling accordingly.

Line 213: The % signs should follow the number without a space.

We thank the reviewer for pointing this out and have removed the spaces before % signs throughout the manuscript.

Line 315: This link 404s: https://github.com/hejDMC/napari-dmc-brainmap This is correct. We are finalizing the manuscript describing the linked tool and the tool is yet to be made public. We plan to make the tool public in the upcoming weeks.

Figure 4: There is a typo throughout ('strenght') We thank the reviewer for catching this error, and we corrected the spelling accordingly.

Reviewer 2 Major comments:

My main concern is the capability of the Raspberry pi to precisely record and control multiple signals simultaneously (the wheel signals, cameras triggering, 2hv camera signal). Indeed, in contrast to single-core devices (such as Arduino or teensy), which operate on a single clock, the Raspberry pi has multiple cores. While this is very interesting for creating complex code or performing higher level of computation, it is generally not recommended for real-time tasks. Indeed, the module RPi.GPIO, used in some of the submitted codes (e.g. sync_pulse.py), seems "[...] unsuitable for real-time or timing critical applications", as described in the library documentation (https://pypi.org/project/RPi.GPIO/). To some extent, this limitation explains why Arduino board are widely used for open-source projects (e.g. PulsePal). Therefore, I would ask the authors to comment and discuss this critical point.

Additionally, the authors may be able to calculate the Raspberry pi 'jitter' for the recorded and sent signals of their setup. By simultaneously recording several signals with a classical DAC (e.g. oscilloscope, national instrument DAC, openEphys or CED) versus a Raspberry, should be feasible and fast to realize such a measure. In my opinion, the interesting t-SNE clustering (figure 5) does not provide a direct proof of the camera synchronization accuracy. However, it suggests that at slow sampling rate, like the one used in this manuscript (100 Hz), synchronization is probably not dramatically affected. Testing different sampling rates could provide further insights and be used as an argument to strengthen the manuscript.

We thank the reviewer for this thoughtful comment. As pointed out by the reviewer, in contrast to single-core devices, Raspberry Pis operate on multiple cores that share the ARM CPU clock whose speed is dynamically adjusted using Dynamic Voltage and Frequency Scaling (DVFS; between 600 MHz to 1.5 GHz). DVFS can contribute to timing variability of CPU-bound operations (e.g. triggering/recording of signals) on Raspberry Pis. Importantly however, all behavioral (i.e. trial data) and time-series data (i.e. rotary data and camera/2p frame pulses) are timestamped and stored individually (see example dataset on the GitHub repository). The timestamps for all datasets are retrieved from the system time (using time.time()) which is not directly influenced by changes in CPU clock speed. Nevertheless, other factors including system overhead, interrupt handling, thread scheduling and garbage collection do impact the timing variability of CPU-bound operations.

We followed the reviewer's suggestion to measure the timing variability ('jitter') for the recorded (input) as well as the sent signals (output). Hence, we performed a simple experiment schematized in Figure R1. In this experiment, we used an Arduino board to generate a 50 Hz pulse train ("2-photon signal") and recorded it using an Open Ephys board and a Raspberry Pi running the auditory detection task. The Raspberry Pi concurrently generated a 20.5 Hz signal ("camera signal") that was simultaneously recorded with the Open Ephys board.

Figure R 1 - Schematic of synchronization test Recording signals (input):

As expected, the timing variability was higher for pulses recorded with the Raspberry Pi than the Open Ephys board (20.0 {plus minus} 2.8 ms vs 20.0 {plus minus} 0.033 ms, Table R1 and Figure R2). Figure R2A shows the recorded timestamps of the Open Ephys Board (x-axis) vs the timestamps recorded by the Raspberry Pi (y-axis; inlets show first and last second of recording, respectively). As apparent, both variables are almost perfectly correlated (R = 1.0) and this correlation is independent of the duration of the recording. Nonetheless, the inter-timestamp intervals recorded by the Raspberry Pi exhibit some degree of 'jitter' (Figure 2RB); however, this variability is minimal, occurring almost exclusively within the single-digit millisecond range (83 out of 14,999 timestamps; 0.55%). Hence, we would like to emphasize that this 'jitter' does not occur on behaviorally relevant timescales and does not significantly impact the alignment of behavioral with time series data (i.e., rotary and 2p/camera imaging data).

Table R 1 - Summary statistics for timing variability (open ephys experiment) Figure R 2 - Synchronization test (input data) Sending signals (output):

We observed similar patterns of timing variability for signals generated and sent by the Raspberry Pi (Table R1; Figure R3A-B). Specifically, our results indicate that Raspberry Pis are less stable than single-core devices (e.g., Arduinos) in producing consistent pulse trains, as evidenced by a mean inter-pulse interval of 48.6 {plus minus} 6.61 ms. Importantly, this variability is captured in the timestamps recorded by the Raspberry Pi itself. Notably, 94.6% of inter-timestamp differences (5842/6173 timestamps) fall within {plus minus}10 ms when comparing Open Ephys and Raspberry Pi recordings (Figure R3C). While this highlights the inherent limitations of Raspberry Pis in maintaining consistent signal timing, it is crucial to emphasize that these fluctuations remain within tolerable bounds for aligning behavioral to e.g. imaging data.

Figure R 3 - Synchronization test (output data) Conclusion for data displayed in Figure 5 Hence, we conclude regarding the data presented in Figure 5 (statistics shown in Table R2; information added to lines 457 and 472) that timing variability ('jitter') is present but occurs at behaviorally irrelevant timescales. Consequently, the alignment of the recorded frames with the behavioral data is not significantly affected. For the camera recordings of the mouse's front paws and face, we estimate a jitter of 34.0 {plus minus} 1.2 ms, demonstrating minimal impact on the accuracy and reliability of the data integration.

The frames of the 2-photon microscope were triggered at 31 Hz, allowing for a comparison of the recorded timestamps with expected timestamps (assuming frames are triggered 'jitterless' at 31 Hz). Here, we find that the vast majority of recorded timestamps (81303/81619 timestamps; 99.61%) deviate by only 0 to 2 ms from the expected timestamps (Table R3), indicating highly consistent timing accuracy.

Table R 2 - Summary statistics for timing variability (Figure 5 data) In summary, timing variability ('jitter') is present but occurs at behaviorally irrelevant timescales (single-digit milliseconds). Importantly, this variability is recorded and does not compromise the alignment of multiple, synchronously recorded time-series with behavioral data. For making this explicit, we added the following discussion of these limitations of the DMC-Behavior Platform to the manuscript's Discussion section (line 516): "A limitation of Raspberry Pis is that e.g. system overhead, interrupt handling, thread scheduling and garbage collection increase the timing variability of CPU-bound operations such as triggering/recording of time-series data (e.g. camera/2-photon frame pulses). We, however, observed that timing variability occurs at behaviorally irrelevant timescales (low, single-digit ms range), without significant effect on the alignment of time-series to behavioral data. Nonetheless, the DMC-Behavior Platform is not suitable for implementing behavioral tasks requiring sub-millisecond precision or recording of frame pulses/timestamps at high sampling rates (> 1 kHz). Instead, we recommend using the platform to generate a (slower) synchronization signal (20-60 Hz) to be concurrently recorded alongside the time-series data (e.g. fiber photometry or electrophysiology). The synchronization signal can be used for post-hoc alignment of time-series to behavioral data (see data presented in Figure 5A-D).

Regarding the reviewer's concerns regarding our interpretation of the t-SNE plots, we agree that the formulation in the manuscript was misleading. The statement has now been changed from "...confirming successful integration..." to "...indicating successful integration..." (line 465).

I would like to emphasize the quality of the manuscript and recommend its publication following the authors' responses to my major comment. I am confident that this manuscript will help researchers working on behavioural experiments customize their methods to fulfill their experimental needs.

Minor comments:

- Line 51: To complete the reference list ("Steinmetz et al., 2021; Zong et al., 2022"), I suggest adding a reference that use large-scale 1photon-miniscope imaging.

We thank the reviewer for raising this point and we have added the following reference:

Ghosh, K. K., Burns, L. D., Cocker, E. D., Nimmerjahn, A., Ziv, Y., Gamal, A. E., &Schnitzer, M. J. (2011). Miniaturized integration of a fluorescence microscope. Nature Methods, 8(10), 871-878. doi: 10.1038/nmeth.1694 - Line 152: Since it is not very common in behavioral training to use ZT4 to ZT6, I would describe the abbreviation 'Zeitgeber Time'.

We agree and thank the reviewer for raising this point. We corrected the manuscript accordingly.

- Line 181: I suggest changing the title to 'Pretraining.' During this phase, the mice associate the cue with the reward. In my opinion, it is a Pavlovian conditioning rather than habituation.

We agree with the reviewer that the term habituation is not used correctly, and we are now using the term 'pretraining' throughout the text.

- Line 190, 208, 211: The authors use the expression "see above". To assist the reader, consider referring to the specific section title in the manuscript.

We thank the reviewer for this observation. We changed the text in the manuscript accordingly.

- Line 233. Could you please describe the abbreviation '#' ' for clarity? We replaced '#'with 'number' in the formula (lines 237).
