# Supplementary material for: The DMC-Behavior Platform: An Open-Source Framework for Auditory-Guided Perceptual Decision-Making in Head-Fixed Mice
Source: eNeuro. 2025 Apr 8;12(4):ENEURO.0457-24.2025. doi: 10.1523/ENEURO.0457-24.2025 (PMC11984799; doi:10.1523/ENEURO.0457-24.2025)
Supplement: Extended data 1 — DMC-Behavior repository. Download Extended data 1, ZIP file. [file eneuro-12-ENEURO.0457-24.2025-s004.zip › tutorial/synchronization_test/Extended Information - Synchronization test.pdf]

Extended information on timing variability using Raspberry Pis

In contrast to single-core devices (e.g. Arduinos), system overhead, interrupt handling, thread scheduling and garbage collection increase the timing variability of CPU-bound operations such as triggering/recording time-series data (e.g. camera/2-photon frame pulses) on Raspberry Pis.

To measure the timing variability ('jitter') for recorded (input) as well as sent (output) signals, we conducted a simple experiment schematized in Figure E1. In this experiment, we used an Arduino Uno R4 Minima to generate a 50 Hz pulse train[1] mimicking the recorded frame pulses from the 2-photon microscope and recorded this pulse train using i) an Open Ephys board and ii) a Raspberry Pi running the auditory detection task (Figure 2). The Raspberry Pi concurrently generated a 20.5 Hz signal like the signal used to trigger camera frames. This signal was recorded with the Open Ephys board.

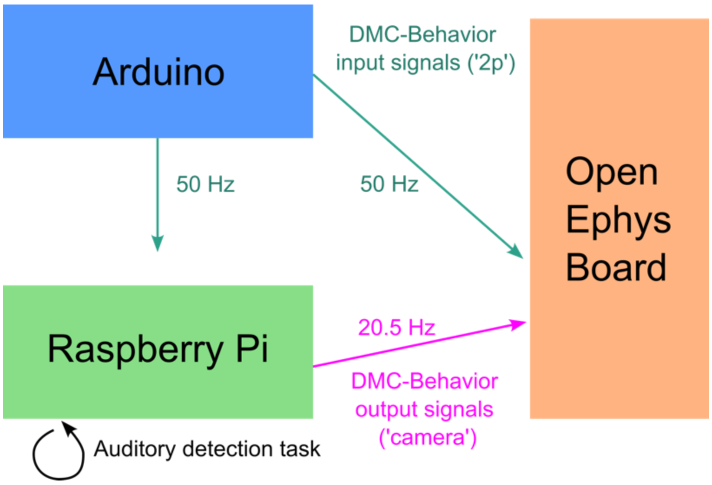

Figure E1 - Schematic of the synchronization test

The results of this synchronization test are summarized in Table E1. As expected, the variability of the recorded (input) timestamps was higher for the Raspberry Pi than the Open Ephys board (20.0 ± 2.8 ms vs 20.0 ± 0.033 ms, Table E1 and Figure E2). Figure E2A shows the timestamps recorded by the Open Ephys board (x-axis) versus the timestamps recorded by the Raspberry Pi (y-axis; inlets show first (top) and last (bottom) second of recording, respectively). We found that timestamps recorded with both devices display an almost perfect correlation (R = 1.0) that is independent of the duration of the recording. Nonetheless, the variability of the timestamps recorded by the Raspberry Pi is higher as shown in Figure E2B, but almost exclusively occurring in single-digit millisecond range (83/14,999 (0.55%)

[1] The Arduino script is located in the same folder as this document.

timestamps display > 10 ms difference). We found similar results regarding the timing variability of timestamps generated and sent (output) by the Raspberry Pi (Table E1 and Figure E3A-B). Specifically, this shows that Raspberry Pis are less stable than single-core devices (e.g., Arduinos) in producing consistent pulse trains, as evidenced by a mean inter-timestamp interval of 48.6 ± 6.61 ms (expected inter-timestamp interval for 20.5 Hz pulse trains is 48.8 ms). Notably, 94.6% of inter-timestamp differences (5842/6173 timestamps) fall within ± 10 ms when comparing Open Ephys and Raspberry Pi recordings (Figure E3C). For the data presented in Figure 5E-G, the recorded 2-photon frame pulses were triggered at 31 Hz, allowing for a comparison of recorded timestamps with expected timestamps (assuming that frames are triggered 'jitterless' at 31 Hz). The results are shown in Table E2. We find that 99.6% (81303/81619 timestamps) of timestamps deviate ≤ 2 ms from the expected timestamps.

In conclusion, Raspberry Pis display increased timing variability both for recording and sending pulses compared to single-core devices such as Arduinos. This fluctuation, however, remain within tolerable limits for aligning imaging to behavioral data and do not occur on behaviorally relevant timescales.

**Recorded signals (input)**		
	Open Ephys Board	Raspberry Pi
Number of frames	15,000	14,999
Frames dropped	0	1
Inter-timestamp-$interval_{mean}$	20.0 ms	20.0 ms
Inter-timestamp-$interval_{Std}$	0.033 ms	2.8 ms
**Sent signals (output)**		
	Open Ephys Board	Raspberry Pi
Number of frames	6173	6173
Frames dropped	0	0
Inter-timestamp-$interval_{mean}$	48.6 ms	48.6 ms
Inter-timestamp-$interval_{Std}$	6.61 ms	6.70 ms

Table E1 - Summary statistics for the synchronization experiment

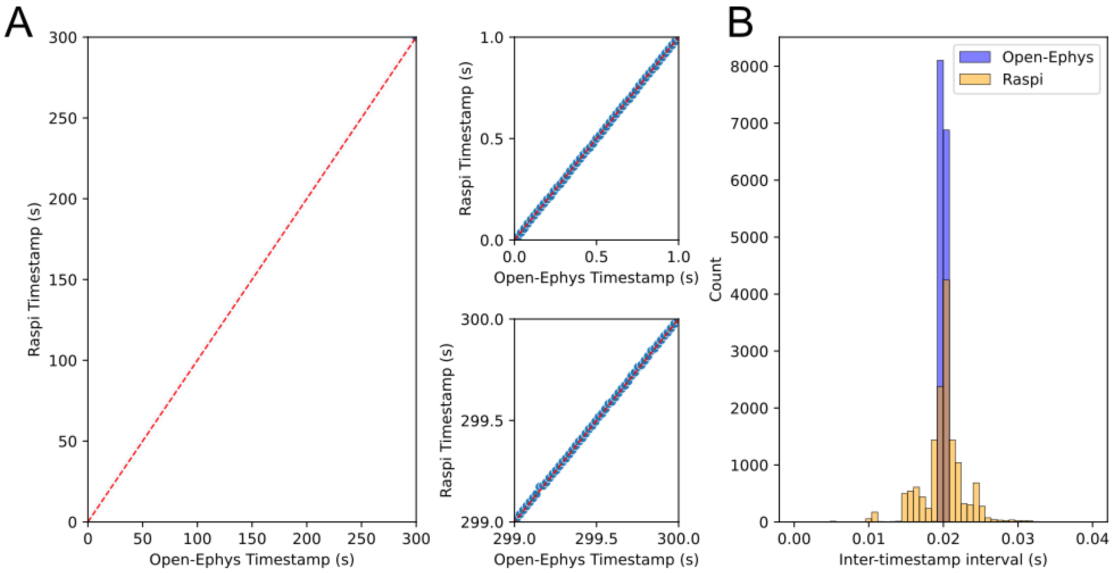

Figure E2 - Synchronization experiment (sending/output)

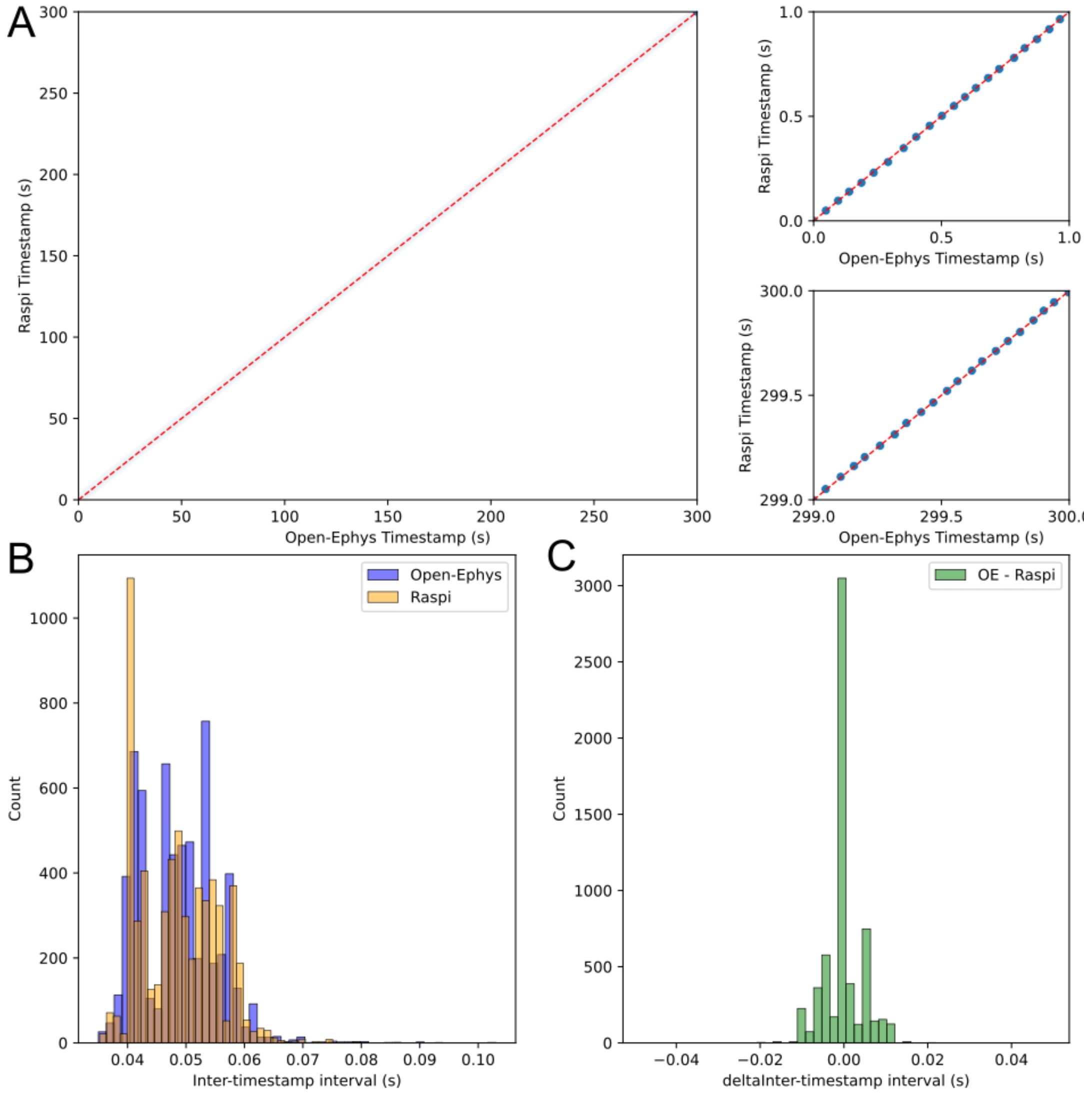

Figure E3 - Synchronization experiment (recording/input)

**Difference range (recorded – expected)**	**Number of timestamps**	**Percentage**
0 to 1 ms	78378	96.03%
1 to 2 ms	2925	3.58%
2 to 3 ms	79	0.10%
3 to 4 ms	51	0.06%
4 to 5 ms	31	0.04%
5 to 6 ms	29	0.04%
6 to 7 ms	24	0.03%
7 to 8 ms	18	0.02%
8 to 9 ms	14	0.02%
9 to 10 ms	10	0.01%
10 to 20 ms	8	0.01%
20 to 30 ms	0	0.00%
30 to 40 ms	17	0.02%
40 to 50 ms	6	0.01%
50 to 100 ms	29	0.04%

Table E2 - Binned count of recorded timestamp deviations from expected timestamps
