# Supplementary figures and images for: The DMC-Behavior Platform: An Open-Source Framework for Auditory-Guided Perceptual Decision-Making in Head-Fixed Mice
Source: eNeuro. 2025 Apr 8;12(4):ENEURO.0457-24.2025. doi: 10.1523/ENEURO.0457-24.2025 (PMC11984799; doi:10.1523/ENEURO.0457-24.2025)

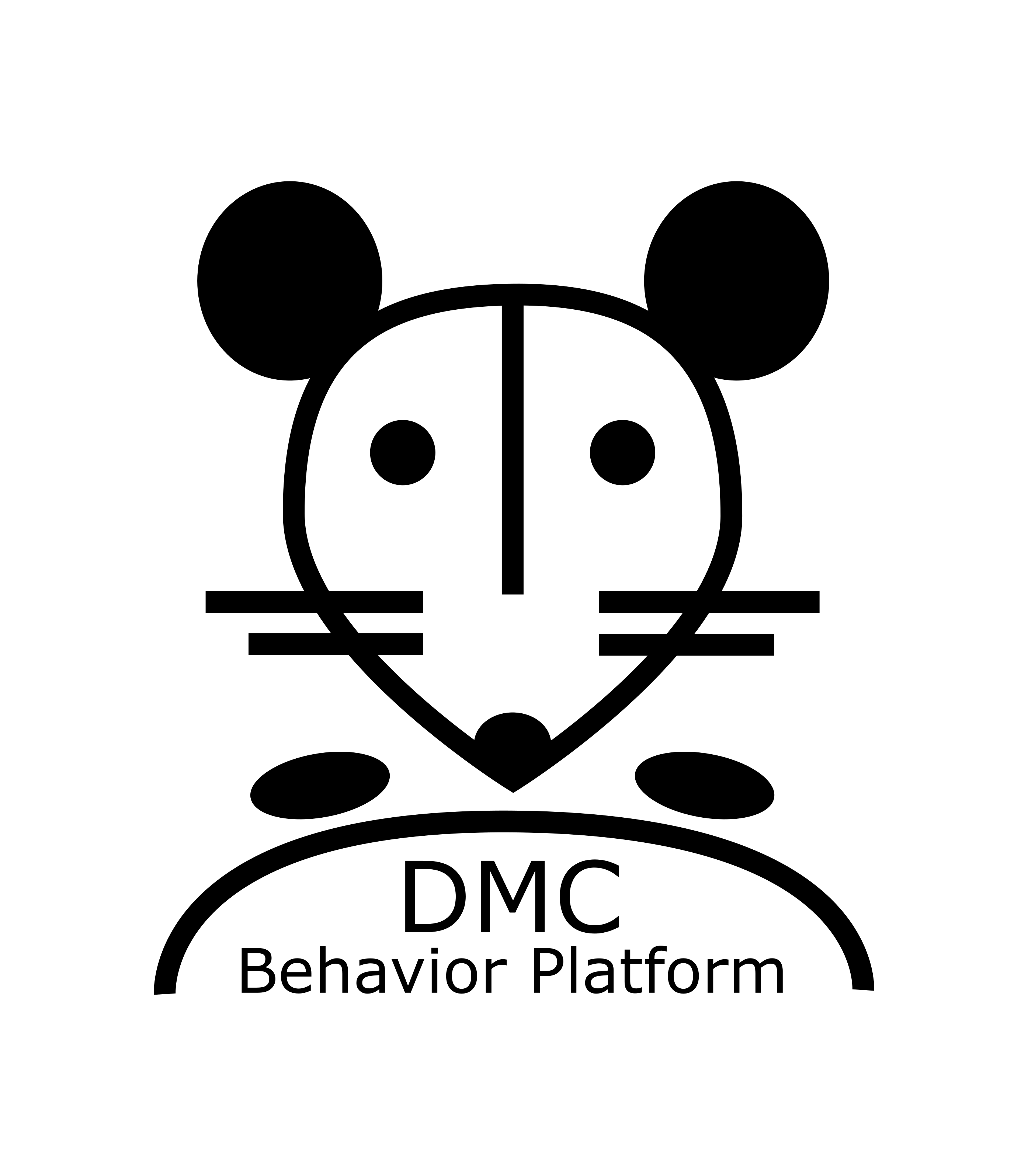

Supplement: Extended data 1 — DMC-Behavior repository. Download Extended data 1, ZIP file. [file eneuro-12-ENEURO.0457-24.2025-s004.zip › docs/logo_dmc-behavior.png]

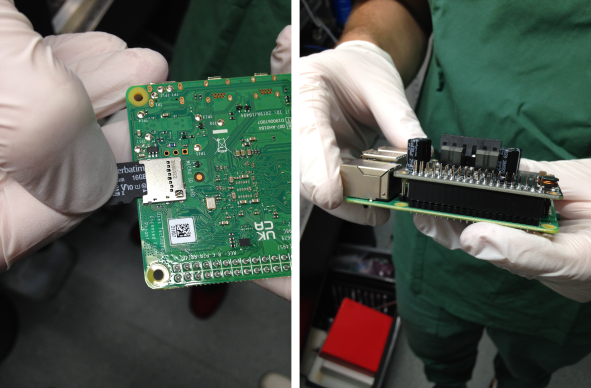

Supplement: Extended data 1 — DMC-Behavior repository. Download Extended data 1, ZIP file. [file eneuro-12-ENEURO.0457-24.2025-s004.zip › docs/pics/hifi.png]

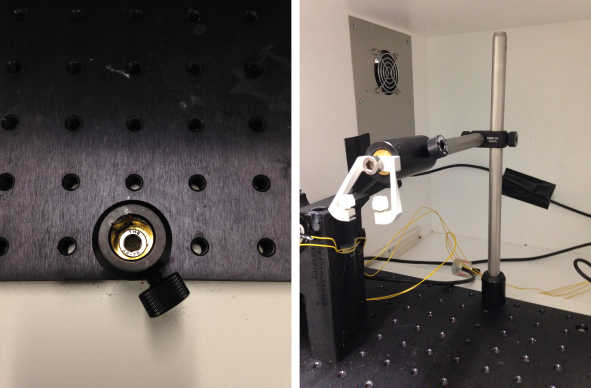

Supplement: Extended data 1 — DMC-Behavior repository. Download Extended data 1, ZIP file. [file eneuro-12-ENEURO.0457-24.2025-s004.zip › docs/pics/holder.png]

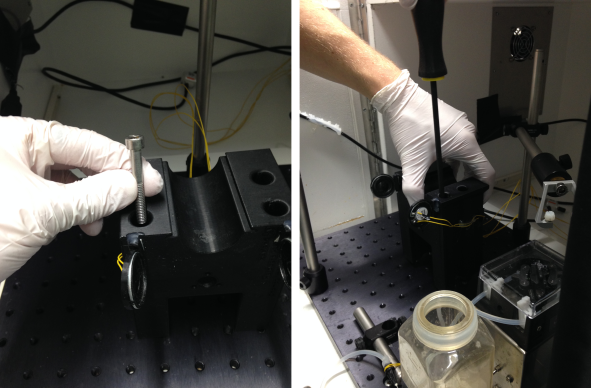

Supplement: Extended data 1 — DMC-Behavior repository. Download Extended data 1, ZIP file. [file eneuro-12-ENEURO.0457-24.2025-s004.zip › docs/pics/mouse-holder.png]

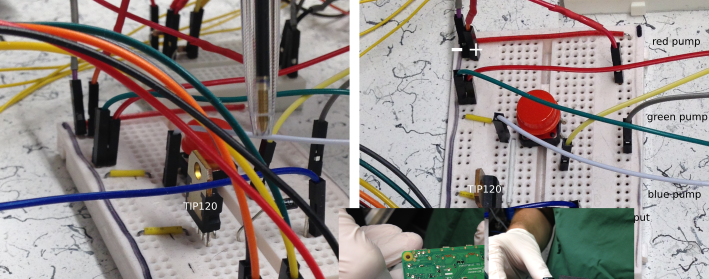

Supplement: Extended data 1 — DMC-Behavior repository. Download Extended data 1, ZIP file. [file eneuro-12-ENEURO.0457-24.2025-s004.zip › docs/pics/pump-wiring.png]

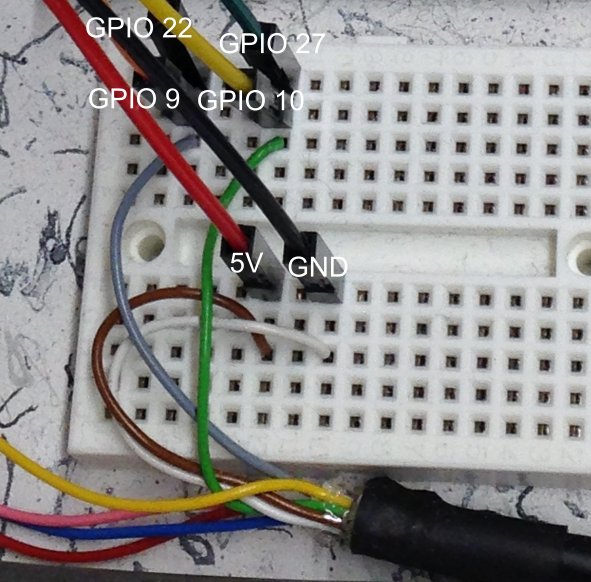

Supplement: Extended data 1 — DMC-Behavior repository. Download Extended data 1, ZIP file. [file eneuro-12-ENEURO.0457-24.2025-s004.zip › docs/pics/rotary.png]

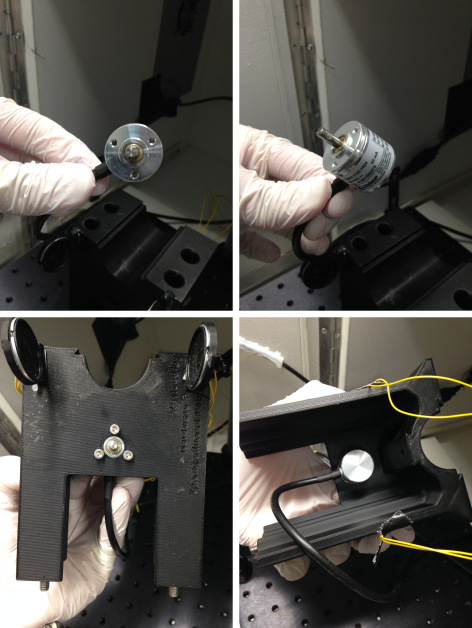

Supplement: Extended data 1 — DMC-Behavior repository. Download Extended data 1, ZIP file. [file eneuro-12-ENEURO.0457-24.2025-s004.zip › docs/pics/rotary_pic.png]

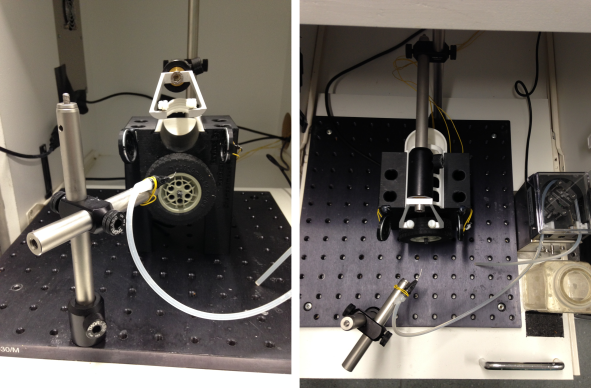

Supplement: Extended data 1 — DMC-Behavior repository. Download Extended data 1, ZIP file. [file eneuro-12-ENEURO.0457-24.2025-s004.zip › docs/pics/setup.png]

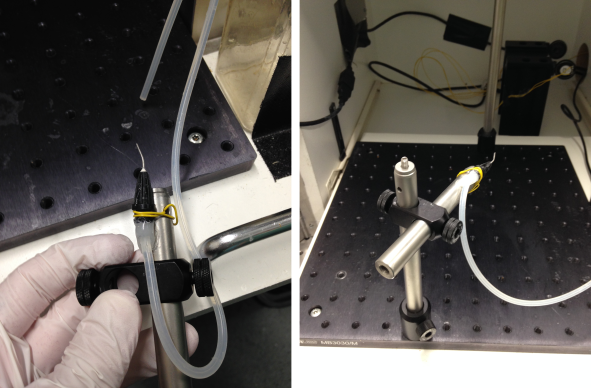

Supplement: Extended data 1 — DMC-Behavior repository. Download Extended data 1, ZIP file. [file eneuro-12-ENEURO.0457-24.2025-s004.zip › docs/pics/spout.png]
